# Physiological function of gut microbiota and metabolome on successful pregnancy and lactation in the captive Yangtze finless porpoise (*Neophocaena asiaeorientalis asiaeorientalis*)^†^

**DOI:** 10.1093/biolre/ioae123

**Published:** 2024-08-13

**Authors:** Syed Ata Ur Rahman Shah, Bin Tang, Dekui He, Yujiang Hao, Ghulam Nabi, Chaoqun Wang, Zhangbing Kou, Kexiong Wang

**Affiliations:** Key Laboratory of Aquatic Biodiversity and Conservation, Institute of Hydrobiology, Chinese Academy of Sciences, Wuhan, China; University of Chinese Academy of Sciences, Beijing, China; Key Laboratory of Aquatic Biodiversity and Conservation, Institute of Hydrobiology, Chinese Academy of Sciences, Wuhan, China; University of Chinese Academy of Sciences, Beijing, China; National Aquatic Biological Resource Center, NABRC, Wuhan, China; Key Laboratory of Aquatic Biodiversity and Conservation, Institute of Hydrobiology, Chinese Academy of Sciences, Wuhan, China; National Aquatic Biological Resource Center, NABRC, Wuhan, China; Key Laboratory of Aquatic Biodiversity and Conservation, Institute of Hydrobiology, Chinese Academy of Sciences, Wuhan, China; National Aquatic Biological Resource Center, NABRC, Wuhan, China; Department of Zoology, Institute of Molecular Biology and Biotechnology, University of Lahore, Lahore, Pakistan; Key Laboratory of Aquatic Biodiversity and Conservation, Institute of Hydrobiology, Chinese Academy of Sciences, Wuhan, China; Key Laboratory of Aquatic Biodiversity and Conservation, Institute of Hydrobiology, Chinese Academy of Sciences, Wuhan, China; Key Laboratory of Aquatic Biodiversity and Conservation, Institute of Hydrobiology, Chinese Academy of Sciences, Wuhan, China

**Keywords:** reproductive stages, microbiome, metabolome, captive Yangtze finless porpoise

## Abstract

Gestation period in captive Yangtze finless porpoise (YFP) is a well-coordinated and dynamic process, involving both systemic and local alterations. The gut microbiota and its connection to fecal metabolites are crucial in supporting fetal development and ensuring maternal health during reproductive stages. This study evaluates changes in the gut microbiota and their correlation with fecal metabolites in captive YFPs during different reproductive stages. The results reveal that microbial community structure changed significantly during reproductive stages, while gut microbial diversity remained stable. The genus unclassified *Peptostrptococcaceae*, *Corynebacterium*, and *norank KD4–96* were significantly greater in non-pregnancy (NP), *Terrisporobacter* was significantly greater in lactating (LL), and *Clostridium* was significantly higher in early-pregnancy (EP) compared to the other groups. The host fecal metabolome exhibited significant alterations during the reproductive stages. Indoxyl sulfate, octadecatrienoic acid, and methionyl-methionine were significantly higher in the NP; galactosylglycerol, chondroitin 6-sulfate, and lumichrome were significantly higher in the EP and mid-pregnancy (MP); and valylleucine and butyryl-l-carnitine were significantly higher in the LL. The altered metabolites were mostly concentrated in pathways associated with arachidonic acid metabolism (significantly altered in NP), leucine, valine, and isoleucine biosynthesis (significantly altered in EP and MP), and glycerophospholipid metabolism (significantly altered in LL compared to others stages). Additionally, we found a strong link between variations in the host metabolism and alterations in the fecal bacteria of captive YFP. In conclusion, this study provides detailed insights into host metabolic and fecal bacterial changes in captive YFP during reproduction stages, providing important knowledge for improving the reproductive management in the captive YFP.

## Teaser

The current study aims to evaluate changes in the gut microbiota and their correlation with fecal metabolites in captive YFPs during different reproductive stages by providing important knowledge for improving the reproductive management of captive YFPs.

## Graphical Abstract

**Graphical Abstract:**
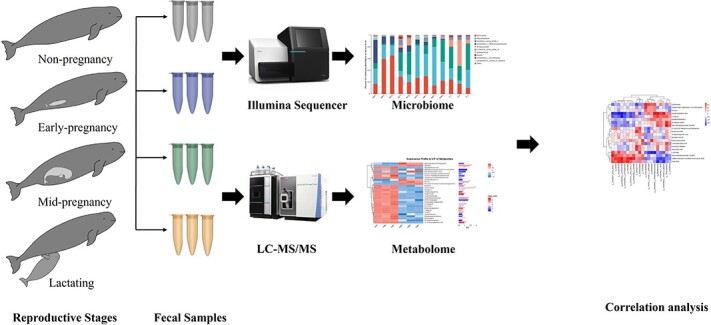

## Introduction

The host’s gut microbiota may help break down non-digestible food sources to improve nutritional and energy absorption [1–3]. The ability of the host to effectively digest food and maintain appropriate nutritional status is directly impacted by alterations in the gut microbiota [2, 4]. The three main reproductive stages for adult female animals are lactation, cycling, and pregnancy. Changes in these reproductive stages affect the energy and nutritional requirements of females [5, 6]. To cope with the energy and nutritional requirements of the fetus and infant, females experience greater nutritional and energy stress throughout pregnancy and lactation. An animal’s daily energy intake increases by 20%–30% during pregnancy and by 37%–39% during breastfeeding compared to the cycling state [7]. Pregnancy and lactation can significantly increase the need for nutrients, such as vitamins, as demonstrated in experimental rats [8]. Both humans and nonhuman primates (NHPs) have a minimum of a one-third increase in their high dietary protein needs during lactation [5]. Reproduction is a costly process for female animals, who bear the majority of the cost of investing in offspring [9]. For the host to be able to satisfy the needs for nutrients and energy throughout pregnancy, recent research has demonstrated that the diversity and community structure of the gut microbiome vary with various stages of pregnancy. For instance, a rise in actinobacteria and proteobacteria may increase the metabolic efficiency of pregnant females, which has also been connected to high levels of inflammation and poor gut microbial diversity [10]. Research on pigs reveals that the gut microbiome’s composition and activity significantly influence the structure of milk during lactation and are strongly linked to host metabolism during pregnancy and lactation [11]. Research on humans during pregnancy has revealed a shift in the gut microbiota’s composition, which may help in immunological and metabolic alterations [10, 12–14]. More recent research has revealed contradictory results, indicating that the gut microbiota remained unexpectedly constant during pregnancy [15–17]. Researchers are utilizing omics technology to study the gut microbiota in more detail, revealing a strong correlation between the intestinal microbiota and animal reproductive success [10]. The gut microbiota influences fetal growth and development as well as maternal metabolic homeostasis through the metabolism of bile acids, hormones, inflammatory factors, and VFAs [18, 19]. Recent research has emphasized the role of gut bacteria in regulating host physiological processes and metabolic changes during pregnancy in humans, sows, and ruminants [10, 11, 20].

The reproductive performance of the critically endangered YFP species, a freshwater cetacean [21], is an important indicator that determines reproduction rates and offspring numbers. The reproductive performance of YFPs is influenced by several aspects, including their health status and feeding management during their reproductive stages. The mother undergoes intricate biological and physiological changes during pregnancy, non-pregnancy, and lactation, which are crucial for her and her infant’s health [22]. Currently, studies on YFP at various reproductive stages mostly focus on blood physiology, biochemistry, hormone indices, and urinary metabolomics due to sample collection challenges and the small number of animals kept in captivity [23, 24]. However, the role of the female intestinal microbiota and its metabolites in regulating physiological metabolic processes in captive YFP’s reproductive stages remains unclear.

## Materials and methods

### Sample collection, animal selection, and husbandry

The study investigated two sexually mature female captive YFPs, YY, and F9. The animals were initially captured in the wild from Poyang Lake in 2009 and 2011, respectively, when they were approximately 2 years old, and were then kept at the Baiji Dolphinarium in Wuhan, China. Due to the unavailability of the fecal samples from the F9 non-reproductive stage and YY pregnancy and lactation stage, the YY samples were used as a control group (NP), which successfully gave birth to a male baby in 2020, and she was just in her reproductive interval stage when F9 was in her pregnancy period. In June 2022, F9 successfully gave birth to a female baby. The fecal samples were collected during her pregnancy and lactation periods.

The main diet of the animals consisted of common carp (*Cyprinus carpio*), sharpbelly (*Hemiculter leucisculus*), and crucian carp (*Carassius auratus*), and the daily intake (about 6%–8% of their body weight per day) was modified by the seasonal variations in the animals’ body mass and appetite. The YFP did not receive any antibiotics or drugs during the sampling process. The water temperature varied seasonally, ranging from 11°C to 27°C. Ivancic et al. [25] defined reproductive success as the birth of a calf that lived for at least ≥30 days. Following the procedures outlined by Hao et al. [24], fecal samples were collected from the captive YFPs during the morning feeding session. Through operant training, the fecal samples were voluntarily taken. The animal was briefly stimulated to turn around its belly in response to the trainer’s behavioral signals. The trainer then used their fingers to softly dab the animal’s genital area. When the animal pooped, the trainer collected it up with a sterilized spoon and immediately reinforced the behavior. The animal’s ID and the date were then written on the centrifuge tube containing the fecal sample. A total of 12 fresh fecal samples were obtained from the four different (d/f) reproductive groups based on the stage of their pregnancy [23]: non-pregnant (NP, before mating), early stage (EP, early four months of gestation), middle stage (MP, mid four months of gestation), and lactating (LL, late lactating stage) and immediately kept at −80°C for analysis of the microbiota and metabolome. According to Zeng et al. [26], the mean gestation period of YFP was calculated to be 349 ± 18 days, or over a year. The YFP mated naturally, and a B-ultrasound was used to diagnose their pregnancy. The Institute of Hydrobiology, Chinese Academy of Sciences, and Research Ethics Committee approved of the study’s methods, which included training animals and collecting fecal samples. The research was carried out strictly in compliance with Chinese regulations and wildlife ethics.

### DNA extraction and PCR amplification

The Quick-DNA TM Fecal/Soil Microbe DNA MiniPrep Kit (Zymo Research) was used to extract the DNA from each fecal sample, following the instructions given by the manufacturer. The bacterial universal primer set consisting of 338F 5′-barcode-ACTCC-TACGGGAGGCAGCA-3′ and 806 5′-barcode-ACTCC-TACGGGAGGCAGCA-3′ was used for amplifying the V3-V4 region of the bacterial 16S rRNA gene. The methods used for PCR amplification and purification were in accordance with earlier research [27]. Briefly, a 20 μl mixture that included 10 ng of DNA template, 4 μl of FastPfu Buffer, 2 μl of dNTPs, 0.8 μl of each primer, 0.4 μl of FastPfu polymerase, and 0.2 μl of BSA was prepared by using the following conditions for the PCR: 3 min at 95°C to allow the DNA to denaturize; 27 cycles of amplification (95°C for 30 s, 55°C for 30 s, and 72°C for 45 s); and a final extension period (10 min at 72°C). A NanoDrop2000 (Thermo Scientific, Wilmington, United States) was used for determining the yield and purity of DNA.

### Sequence processing and bioinformatics analysis

Using the Illumina MiSeq PE300 platform (Illumina, San Diego, USA), 16S rRNA gene sequencing was performed in fecal samples to determine the composition of the gut microbiota. Majorbio Bio-Pharm Technology Co. Ltd. (Shanghai, China) conducted paired-end sequencing on purified amplicons in an equimolar ratio. The Majorbio Cloud platform (https://cloud.majorbio.com/) has been used for the bioinformatics analysis of the gut microbiome. The alpha diversity index, which includes Chao1, ACE, Simpson, Shannon, and good coverage and is based on the OTUs dataset, is computed using Mothur v1.30.1. The Vegan v2.5-3 package was used for a principal coordinate analysis (PCoA) and PERMANOVA test to analyze microbial communities in various samples based on Bray–Curtis dissimilarity and statistical significance of the variance. The study utilized linear discriminant analysis (LEfSe) (http://huttenhower.sph.harvard.edu/LEfSe) to identify the numerous bacteria taxa across different groups, with an LDA score > 2.0, *P* < 0.05 [28]. The Kyoto Encyclopedia of Genes and Genomes (KEGG) database was used to determine functional conclusions, while PICRUSt2 was used to predict metabolic gene profiles, following procedures outlined on GitHub (https://github.com/picrust/picrust2/wiki) [29]. The statistical differences between the groups were calculated at the phylum and genus levels using the Wilcoxon rank-sum test [30] and the Benjamini–Hochberg (BH) method was used to adjust *P*-values [31]. R version 4.1.2 was used to perform and illustrate subsequent analysis and plotting.

### Untargeted metabolomics analysis

Fecal metabolites in the d/f reproductive stages were identified using a non-targeted LC–MS/MS metabolomics approach. In general, weigh a 100 μl fecal sample precisely and extract metabolites using 400 μl of a 4:1 v/v methanol/water solution. The mixture was allowed to settle at around −20°C before being processed for six minutes at 50 Hz using a high-throughput tissue crusher (Wonbio-96c, Shanghai Wanbo Biotechnology Co., Ltd.). Following that, it was vortexed for 30 s and then put through 30 min of 40 kHz, 5°C ultrasonic treatment. To precipitate proteins, samples were kept at −20°C for 30 min. Following centrifugation at 13 000 g for 15 min at 4°C, the supernatant was carefully transferred into sample vials for LC–MS/MS analysis. After LC–MS/MS analysis, peak identification and alignment were performed using the raw data that was loaded into Progenesis Q1 2.3 (Nonlinear Dynamics, Waters, USA). A data matrix including the retention time (RT), mass-to-charge ratio (m/z), and peak intensity was generated as a result of preprocessing. At least 80% of the metabolic characteristics remained in each sample group. Summation was used to normalize each metabolite characteristic following filtering. The minimum metabolite value for samples with metabolite levels below the quantitative lower limit was determined. Metabolite characteristics having a relative standard deviation (RSD) of QC > 30% were excluded from data QC using internal standards. Accurate mass, MS/MS fragment spectra, and isotope ratio differences were identified using reliable biochemical databases like Metlin (https://metlin.scripps.edu) and the Human Metabolome Database (https://www.hmdb.ca/). We conducted a multivariate statistical analysis on the Majorbio Cloud Platform (https://cloud.majorbio.com), using the R package ropls (Version 1.6.2, http://bioconductor.org/packages/release/bioc/html/ropls.html) from Bioconductor.

### Statistical methods and data processing

The relationship between metabolites and the abundance of the gut microbiome (genera) was investigated using the Spearman rank correlation coefficient. A coefficient greater than 0.7 and a *P*-value of less than or equal to 0.05 were used to select plots for each metabolite.

## Results

### Sequencing metadata

Twelve fecal samples (3 each from non-pregnancy, early pregnancy, mid-pregnancy, and lactating stages) were sequenced using Illumina MiSeq, producing 732 462 (mean: 59 602; range: 53 598–72 188) raw 16S rDNA reads, resulting in 826 OTUs. For each sample, a saturation plateau was attained by the Shannon rarefaction curve between the number of reads and the Shannon index at the OTU level (Supplementary Figure S1a). The analyses identified were assigned to 1 kingdom, 13 phylum, 23 classes, 59 orders, 83 families, 117 genera, 153 species, and 826 OTUs. The top five phylum-level species include Firmicutes, Actinobacteria, Unclassified bacteria, Cyanobacteria, and Proteobacteria, while the top five genus-level species include *Romboutsia*, *Paeniclostridium*, *Clostridium_sensu_stricto_1*, *Unclassified Peptostreptococcaceae*, and *Terrisporobacter*.

### The gut microbiota’s composition

The distribution of OTUs in the bacterial community was shown in the Venn diagram (Supplementary Figure S1a). The study found 259, 208, 188, and 171 OTUs in the NP, EP, MP, and LL groups, with NP sharing a bacterial community of 48 OTUs with EP, MP, and LL.

### Alpha diversity analysis in the gut microbiota

The ACE richness estimator and Chao index in NP were higher than those in EP, MP, and LL groups, based on the results of the alpha diversity analysis using the Wilcoxon rank sum test at the OTU level, as shown in Supplementary Table S1 and Supplementary Figure S2a, and b. The Shannon index was greater in LL and EP than in MP and NP (Supplementary Figure S2c), while the Simpson diversity index in NP and MP was greater than that in EP and LL (Supplementary Figure S2d). The Ace, Chao, Simpson, and Shannon indices showed no significant difference (*P* > 0.05), indicating minimal change in community diversity among the four groups.

### Beta diversity analysis in the gut microbiota

**Figure 1:**
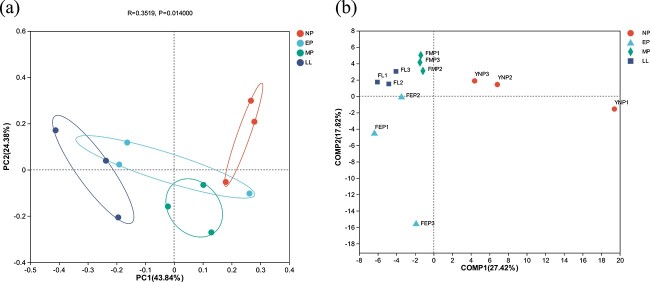
The PCoA using (a) Bray–Curtis distance and (b) PLS-DA of OTUs revealed variations in fecal bacteria community structures of captive YFP during different reproductive stages.

The Bray-Curtis methods were utilized to conduct principal coordinated analysis (PCoA) of beta diversity, analyzing compositional differences in fecal microbiota among four groups (Figure 1a–b). The study found significant differences among the four groups based on Bray-Curtis distances (ANOSIM, *P* < 0.05). The PLS-DA method was employed to analyze the high-dimensional data and differentiate observed values among different groups (Figure 1b). In the NP, EP, MP, and LL samples, the fecal bacterial communities were grouped apart.

### Analysis of gut microbiota species composition and differences

**Figure 2:**
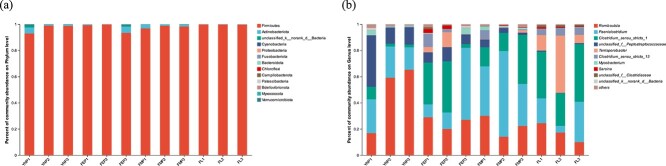
The study reveals differences in gut microbiota abundance between four d/f reproductive groups at (a) phylum and (b) genus levels, with a histogram illustrating the relative abundance of the top 10 bacterial phyla and genera in the captive YFP.

The composition of the gut microbiota in four d/f groups is shown in Figure 2. The main annotated groups were Firmicutes (97, 98, 98, and 100%), Actinobacteria (2, 2, 2, and 0%), and Unclassified bacteria (1, 0, 0, and 0%), in the NP, EP, MP, and LL groups, respectively (Figure 2a) at the phylum level. *Romboutsia* (47, 25, 22, and 17%), *Paeniclostridium* (22, 26, 45, and 18%), *Clostridium_sensu_stricto_1* (5, 25, 22, and 35%), *Peptostreptococcaceae* (22, 9, 4 and 1%), *Terrisporobacter* (0,5 1, 21%), *Clostridium_sensu_stricto_13* (0, 4, 3, and 5%), *Mycobacterium* (2, 2, 2, and 0%), *Sarcina* (0,2,0, 1%), *Clostridiaceae* (0,1,0, 1%), and *Epulopiscium* (0, 1, 1, 0%) were the abundant bacterial genera of captive YFP in the four d/f groups, respectively (Figure 2b). The gut microbiota of the captive YFP varied considerably with the d/f reproductive stages, based on the above analysis of species composition and community structure.

### Bacterial biomarkers of different pregnancy stages

**Figure 3:**
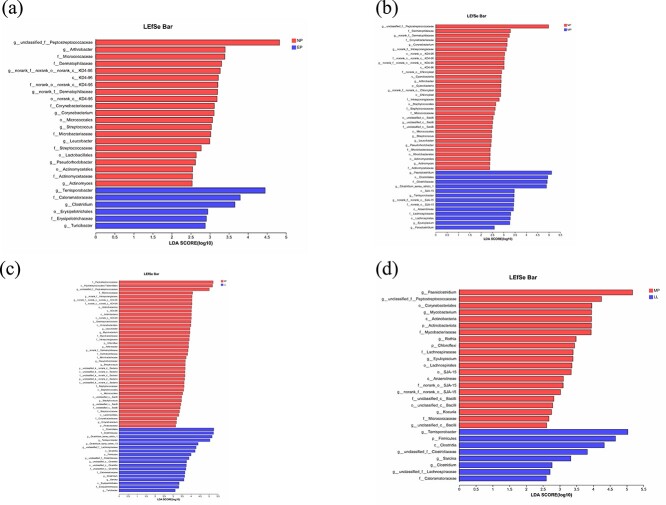
The histogram shows the LDA scores for differentially abundant fecal bacteria among d/f reproductive groups in the captive YFP, i.e. (a) NP vs. EP, (b) NP vs. MP, (c) NP vs. LL, (d) MP vs. LL, with LDA > 2, *P* < 0.05.

The LEfSe analysis was utilized to identify microorganisms with significant relative abundance variations across four groups, based on the effect pattern of LDA (LDA > 2, *P* < 0.05). The abundance of bacteria, which is significantly different in the four reproductive groups, is shown in Figure 3a-d. The study found that the dominant species in fecal samples from the NP, compared to EP, MP, and LL, were from the genus unclassified *Peptostreptococcaceae*, *norank KD4–96*, and *Arthrobacter*, while in the EP, MP, and LL were dominant by *Terrisporobacter*, *Clostridium*, *Turicibacter*, and *Paeniclostridium*. The genus *Paeniclostridium*, unclassified *Peptostreptococcaceae*, and *Rothia* were dominant in the MP compared with LL, which was dominant by *Terrisporobacter* and *Clostridium*.

### Potential functional capacity of the captive YFP fecal microbiome during reproductive stages

The study utilized PICRUSt software to analyze the potential functional capacity of the captive YFP fecal microbiome using 16S rRNA data. The study compared the relative abundances of KEGG pathways among different reproductive groups, finding no significant difference in the top 50 pathways (Supplementary Figure S3).

### Comparison of the fecal metabolome in captive YFP in d/f reproductive stages

**Figure 4:**
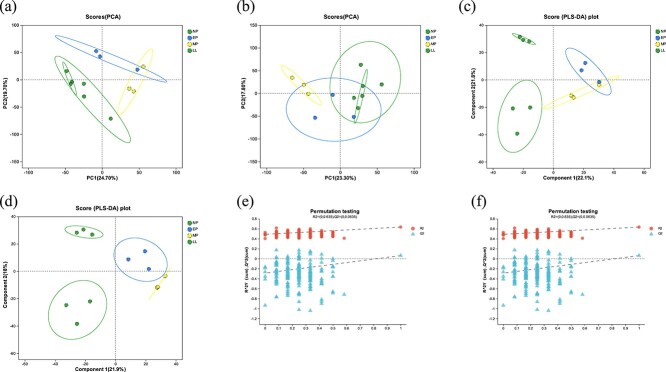
Analysis of the metabolomics data’s quality of the captive YFP. Samples obtained in the positive ion mode (a) and negative ion mode (b) are shown according to their PCA scores. The confidence ellipse indicates 95% confidence in the distribution of “real” samples, with higher separation degrees indicating a more significant classification effect. The positive ion mode (c) and the negative ion mode (d) are shown according to their PLS-DA score plots. The abscissa represents the permutation reservation test, while the ordinate represents the substitution tests for R2 and Q2, and the two dotted lines represent the regression lines for R2 and Q2. The positive ion mode (e) and the negative ion mode (f) are shown according to their PLS-DA permutation test, respectively.

In this study, in the positive ionization mode, a total of 1539 metabolites were identified, while in the negative ionization mode, a total of 751 metabolites were identified following annotation (Supplementary Table S2). In addition, strong clustering was seen in the QC samples near the plot’s origin, suggesting that the instrument had high reproducibility and stability. The unsupervised PCA model distinguished metabolites in four groups (Figure 4a and b), with PLS-DA results indicating distinct metabolites (Figure 4c and d). According to the PLS-DA model, the model fitted well, was highly predictable, and was suitable for data analysis (Figure 4e and f). The intercepts of the regression line of Q2 and vertical axis *Y* were 0.162 and 0.0635, respectively. The R2 in the positive and negative ion models was also higher than Q2.

### Potential differential metabolites in captive YFP during reproductive stages

**Figure 5:**
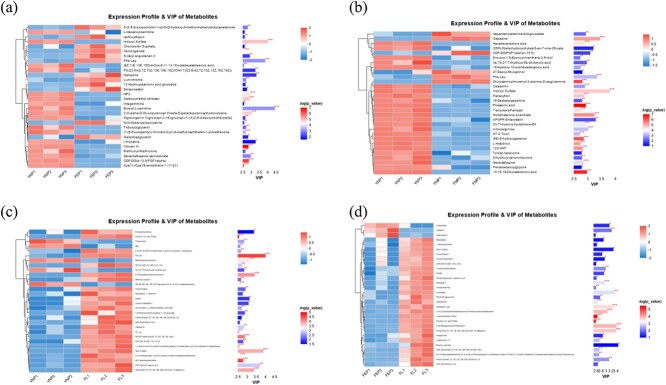
Screening of potential differential metabolites in the captive YFP. (a-d) The differential metabolites of captive YFP were analyzed using cluster analysis in the NP-EP, NP-MP, NP-LL, and EP-LL, respectively. The metabolites’ clustering dendrogram is presented on the left. The expression patterns of all metabolites in the sample show greater similarity as the branches are closer together. The metabolite VIP bar chart is on the right side, with the bar’s length representing its contribution to the variation between the two groups. The bar’s color indicates the significance of the difference between the two groups, with larger log10 values indicating smaller p-values and darker colors. The symbol “^*^” indicates *P* < 0.05, “^*^^*^” *P* < 0.01, and “^*^^*^^*^” *P* < 0.001.

The OPLS-DA model was used to obtain the VIP. The four reproductive groups’ DMs were subsequently identified using VIP > 1.0 and a *P*-value <0.05 (Figure 5 a-d). A total of 1434 DMs in both the positive and negative ion modes were identified (Supplementary Table S3, Supplementary Figure S4). As shown in (Figure 5) compared with NP-EP, 316 DMs were identified; among them, 152 DMs (dexamethasone isonicotinate, vincristine, integerrimine, l-hexanoylcarnitine, indoxyl sulfate, carboxymethyl chitosan, octadecatetraenoic acid, methionyl-methionine, and butyryl-l-carnitine) were upregulated in the NP, and 164 DMs (gabazine, doisynoestrol, galactosylglycerol, chondroitin 6-sulfate, phe leu, lumichrome, 5-valyl angiotensin II, and 12-hydroxyjasmonic acid glucoside) were down-regulated in the EP. Compared with NP-MP, 479 DMs were identified; among them, 300 DMs were upregulated in the NP (tyrosyl-isoleucine, 16-hydroxy-10-oxohexadecanoic acid, dihydroxylysinonorleucine, indoxyl sulfate, l-histidinol, and phaseolic acid), and 179 DMs (isopentenyladenine-9-n-glucoside, phe leu, gabazine and glucosaminylmuramyl-2-alanine-d-isoglutamine) were down-regulated in the MP. Compared with NP-LL, 358 DMs were identified; among them, 121 DMs (dihydroxylysinonorleucine, triterpenoids, and (6Z,9Z,12Z,15Z,18Z,21Z)-tetracosa-6,9,12,15,18,21-hexaenoylcarnitin) were up-regulated in the NP, and 237 DMs (aplysiatox, alarelin, cyclotricuspidoside c, prexanthoperol, methionyl-arginine, phe leu, valylleucine, renin inhibitor, and tyr leu) were down-regulated in the LL. Compared with EP-LL, 281 DMs were identified; among them, 98 (cinobufotalin, gabazine, and doisynoestrol) DMs were up-regulated in EP, and 183 DMs (mesulergine, l-hexanoylcarnitine, renin inhibitor, prexanthoperol, alarelin, nodulisporic acid, PS(20:2(11Z,14Z)/TXB2), integerrimine, virginiamycin m1, butyryl-l-carnitine, and PA(8:0/22:6(4Z,7Z,11E,13Z,15E,19Z)-2OH(10S,17)) were down-regulated in the LL.

### Metabolic pathway enrichment analysis

**Figure 6:**
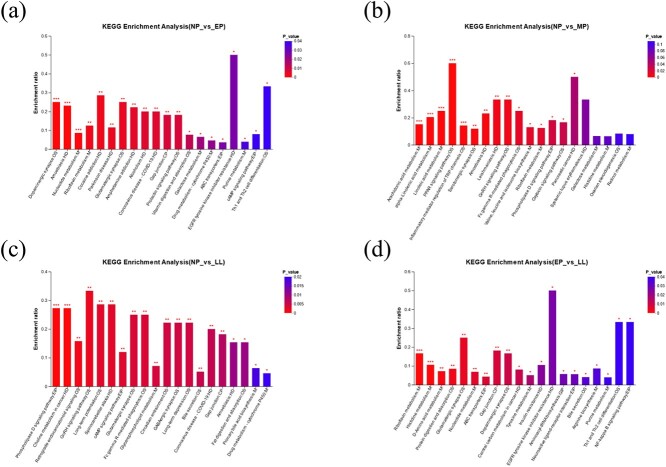
Differential metabolites’ enrichment in metabolic pathways between (a) NP vs. EP, (b) NP vs. MP, (c) NP vs. LL, and (d) EP vs. LL, in the captive YFP. ^*^P < 0.05, ^*^^*^P < 0.01, ^*^^*^^*^P < 0.001.

The metabolites that were found were categorized into second-grade KEGG pathways. The top searches for the term “metabolism” were amino acid metabolism, metabolism of cofactors and vitamins, xenobiotics biodegradation and metabolism, carbohydrate metabolism, lipid metabolism, nucleotide metabolism, metabolism of other amino acids, energy metabolism, and biosynthesis of other secondary metabolites (Supplementary Figure S5). The KEGG metabolic pathways that show a *P* < 0.05 are considered to be the most significant pathways involved in this study (Figure 6a-d; Supplementary Table S4). The NP and EP groups have significantly different metabolic pathways such as nucleotide metabolism, riboflavin metabolism, glutamatergic synapse, prolactin signaling pathway, vitamin digestion and absorption, galactose metabolism, ABC transporters, and purine metabolism. The results of the enrichment analysis indicated a substantial difference between the NP and MP groups in terms of metabolic pathways, including alpha-linolenic acid metabolism, arachidonic acid metabolism, linoleic acid metabolism, inflammatory mediator regulation of TRP channels, serotonergic synapse, valine, leucine and isoleucine biosynthesis, and riboflavin metabolism. The metabolic pathways of the GnRH signaling pathway, glutamatergic synapse, glycerophospholipid metabolism, bile secretion, fat digestion and absorption, and primary bile acid biosynthesis were significantly different in the NP and LL groups. Protein digestion and absorption, riboflavin metabolism, D-amino acid metabolism, histidine metabolism, ABC transporters, nucleotide metabolism, arginine biosynthesis, tyrosine metabolism, purine metabolism, and bile secretion were significantly altered between the EP and LL groups.

### Correlation analysis between the gut microbiome and metabolite profiles

**Figure 7:**
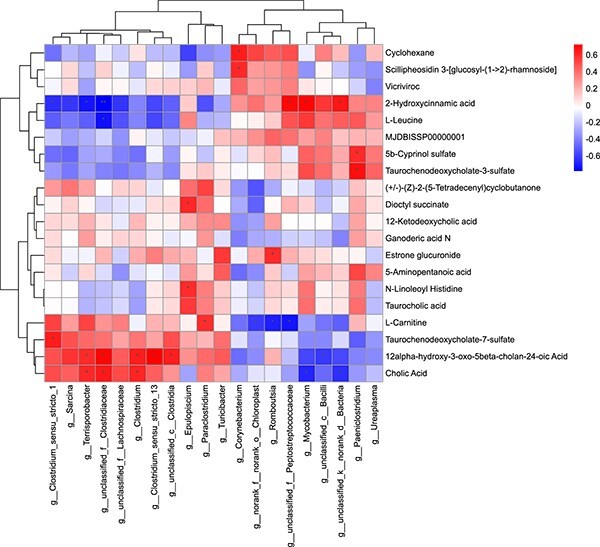
Multi-omics approaches revealed microbiota-metabolite interactions in the captive YFP in d/f reproductive groups. Heatmap showing the relationship between gut microbiota and metabolite clusters. Asterisks represent statistically significant correlations (*P* < 0.05) between microorganisms and metabolite clusters; ^*^,^*^^*^, and ^*^^*^^*^ represent statistically significant differences (*P* < 0.05, *P* < 0.01, and *P* < 0.001, respectively).

A Spearman correlation analysis was conducted on 20 metabolites and microbes, revealing a positive correlation between *Clostridium_sensu_stricto_1*, *Terrisporobacter*, *unclassified Clostridiaceae*, *Clostridium*, *Clostridium_sensu_ stricto_13*, *unclassified Clostridia*, *Epulopiscium*, *Paraclostridium*, *Corynebacterium*, *Romboutsia*, *unclassified Peptostreptococcaceae*, *Mycobacterium*, *unclassified bacteria,* and *Paeniclostridium* was positively correlated with taurochenodeoxycholate-7-sulfate, cholic acid, 12alpha-hydroxy-3-oxo-5beta-cholan-24-oic acid, dioctyl succinate, n-linoleoyl histidine, l-carnitine, cyclohexane, scillipheosidin 3-[glucosyl-(1- > 2)-rhamnoside], estrone glucuronide, 2-hydroxycinnamic acid, 5b-cyprinol sulfate and taurochenodeoxycholate-3-sulfate, while *Clostridium_sensu_ stricto_1*, *Sarcina*, *Terrisporobacter*, *unclassified Clostridiaceae*, *unclassified Lachnospiraceae*, *norank Chloroplast*, *Romboutsia*, *unclassified Peptostreptococcaceae*, *Mycobacterium*, *unclassified bacteria*, *unclassified Bacilli* were negatively correlated with 2-hydroxycinnamic acid, l-leucine, l-carnitine, cholic acid, and 12alpha-hydroxy-3-oxo-5beta-cholan-24-oic acid, as shown in (Figure 7). The Spearman correlation coefficient’s size is shown by different colors. A positive correlation is indicated by red, and a negative correlation is indicated by blue.

## Discussion

Marine mammals, such as cetaceans, require long gestation periods to produce fully developed offspring [32, 33]. According to Zeng et al. [26], the mean gestation period of YFP was estimated to be 349 ± 18 days or almost a year. A comprehensive knowledge of the reproductive physiology of cetaceans is required to ensure their successful conservation in the wild [34, 35]. However, due to the limitations of their aquatic habitat, research on cetaceans is challenging [35]. During the reproductive stages, the maternal gut microbiota and metabolites may have an impact on maternal health and fetal development. The present research showed changes in fecal microbiota and metabolites during pregnancy.

The study reveals that fecal bacteria in captive YFP vary among different reproductive stages, but there was no significant alteration in the four d/f groups. The diversity of the gut microbiota is a significant indicator of health and metabolic capacity, and it is well recognized to enhance the performance and stability of communities [36–38]. Maternal physiological metabolism changes during various reproductive stages, with factors like uterine growth, gastrointestinal tract contraction, peristalsis, and hormone fluctuations altering the structure of the gut microbiota [10, 39]. Thus, diverse bacteria likely offer functional redundancy and numerous metabolic capabilities during YFP pregnancy, providing essential nutrients for fetus growth and the mother’s health. The study found significant variations in bacterial composition across different reproductive stages, as evidenced by beta-diversity results from PLS-DA and NMDS analyses. The results were aligned with other research that reported significant alterations in the microbiome composition throughout gestation [40–42].

However, it is widely accepted that a variety of host and environmental factors can impact the gut microbiota [43]. The study of gut microbiota alterations is challenging due to the inability to account for host genetics, nutrition, and individual variations [44–46]. In the current study, the samples were collected from two animals with almost the same background and demonstrated significant variability in different reproductive stages, even from the same individual. The current study found that Firmicutes were dominant in the captive YFP fecal samples, consistent with previous research by Freetly et al. [47], who found that Firmicutes accounted for the majority of bacteria found in the duodenum (86%) and jejunum (99%) of beef cattle, and Guo et al. [48], who found 75% of Firmicutes abundance in d/f reproductive stages in mice. This finding supports the importance of gut bacterial populations in affecting host health and functionality [49]. The abundance of Firmicutes remained stable throughout the reproductive stages, consistent with previous research that found no significant changes during human pregnancy [10, 50]. Changes in the relative abundance of specific bacterial taxa are generally associated with dietary components [51]. Therefore, no variations in Firmicutes were detected in the current study when the captive YFP were fed with the same and constant diet (unpublished work). Actinobacteria were abundant in the NP, EP, and MP as compared to the LL, which is in accordance with the increase of Actinobacteria from the first to the third trimester during pregnancy in humans [10], which is beneficial for fetal growth and energy demands during lactation.

*Clostridium sensu stricto 1* was abundant in the LL as compared to the other stages due to their high food diet and high energy requirements [52]. It has been shown that *Clostridium sensu stricto 1* utilizes mucus-derived saccharides as an energy source to produce SCFAs and to strengthen the intestinal mucus barrier, which prevents pathogen adhesion [53]. *Peptostreptococcaceae* was significantly higher in the NP as compared to the other groups. Diet appears to have a significant impact on the *Peptostreptococcaceae* genus [54, 55]. For instance, when dietary protein intake decreased from 16% to 13% in adult pigs, the relative abundance of *Peptostreptococcaceae* increased [54]. In addition, when calories were restricted, mice provided with a high-fat or low-fat diet had lower *Peptostreptococcaceae* [55]. An anaerobic pathogen, *Terrisporobacter*, has been proven to induce oxidative stress [56, 57]. *Terrisporobacter* was significantly more abundant in the LL stage than in the other groups, but unexpectedly, it was less abundant in the MP than in the EP, which is consistent with research by Zhou et al. [58] and Chen et al. [59], which also revealed decreased levels of *Terrisporobacter* in the feces of pregnant sows. The decreased level of *Terrisporobacter* during pregnancy may relieve oxidative stress and improve the maternal health of the captive YFP. In EP and MP, the genus *Epulopiscium* was more numerous than in NP and LL, which also increased during pregnancy in sows [44]. The genus *Clostridium*, which is a member of the phylum firmicutes and is involved in the decomposition of cellulose, was significantly increased in the EP when compared to the other groups and reported to be increased in pregnant pandas, while reported to be lowered in the preterm group as compared to full-term pregnant humans [60–62]. *Clostridium* induces T-cells in mice, which can prevent preterm birth by inhibiting inflammation through interleukin-10 production [60]. So, the higher abundance of *Clostridium* during pregnancy may be beneficial for the healthy pregnancy of the captive YFP.

The PICRUSt predicts no significant variation in the gut microbiome’s functional capacity among reproductive stages in captive YFP, indicating no significant changes in the main phylum Firmicutes. The top five metabolic functions of bacteria were energy, lipid, cofactor, vitamin metabolism, amino acid metabolism, and carbohydrate metabolism. The captive YFP gut microbiota’s functional capacity remained relatively constant throughout the reproductive stages, likely providing sufficient nutrients for the mother’s health and the fetus’s development.

This study is the first to comprehensively examine fecal metabolic changes in captive YFP during reproductive stages using the LC–MS-based metabolomics technique. When compared to the NP, the metabolites were more abundant in pathways associated with the metabolism of alpha-linolenic acid, arachidonic acid, linoleic acid, vitamin absorption and digestion, valine, leucine, and isoleucine, riboflavin metabolism, nucleotide metabolism, glucose metabolism, glycerophospholipid metabolism, and purine metabolism during the reproductive stages.

Organic acids such as indoxyl sulfate were significantly higher in the NP as compared to the EP, which was reported to be significantly higher in the infertile ewe [63]. Indoxyl sulfate, a byproduct of tryptophan metabolism, is primarily produced by gut microflora [63, 64] and supports mixed microbial communities’ survival. It increases oxidative stress and inflammation in macrophages and the intestinal mucosa [65–67]. The fatty acid octadecatrienoic acid is a precursor to various hormone-like compounds, including thromboxane, prostacyclin, prostaglandin, and leukotriene. These compounds, which were higher in the NP than in the other reproductive stages, have an impact on immune function, wound healing, nervous system stimulation, improved embryo quality, and fertilization in non-super-ovulated lactating dairy cows [68, 69]. The amino acid methionyl-methionine was significantly higher in the NP compared to the EP. An essential amino acid for the production of proteins is methionine. In a mouse model of methionine deficiency, a partial supply of methionine during pregnancy could improve reproductive performance over methionine alone, suggesting that decreased methionine availability during pregnancy may impair intestinal function due to active regeneration in the intestine [70]. In the EP, compared to the NP, there was a significant rise in lipids and lipid-like compounds called galactosylglycerol. These molecules are essential for the metabolism of carbohydrates and galactose as well as fetal development. Additionally, it was reported that pregnant women had a high level of galactosylglycerol [71]. The EP group revealed a significantly higher amount of the organic oxygen compound chondroitin 6-sulfate in comparison to the NP group. This molecule is essential for the morphogenesis of mammalian cartilage and embryonic development [72]. Organoheterocyclic compounds such as lumichrome, which are essential for the metabolism of cofactors and vitamins as well as riboflavin, were found in significantly higher concentrations in the EP compared to the NP. The present findings are consistent with the research conducted by Li et al. [73], indicating elevated levels of lumichrome during pig gestation. Norethisterone enanthate, a lipid and lipid-like molecule that is greater in the NP compared to the MP and is used as a contraceptive in both males and females, is said to suppress the mid-cycle surge of FSH and LH [74]. Triterpenoids, which are enriched in the NP compared to the LL stage, are among the physiologically active substances (corticosteroids, sex hormones, etc.) that control vital processes (organism-coordinated growth, differentiation, and reproduction) in vertebrates and humans [75]. Valylleucine, an organic acid, was substantially greater in LL compared to NP. This difference may be attributed to the healthy eating index, which is linked to better metabolic function in humans [76]. The primary metabolite in buffalo milk has been reported to be butyryl-l-carnitine [77], which was shown to be substantially higher in the LL as compared to other reproductive stages. Intestinal inflammation may be treated by butyryl-l-carnitine [78]. In the liver, desaturation, chain elongation, and metabolic conversion result in the formation of arachidonic acid (AA). Because the fetal liver’s ability for desaturation and chain-elongation is immature in the early stages of gestation in both pigs and humans, the fetus is mostly dependent on the mother for a supply of pre-formed AA [79, 80]. The metabolism of AA varied significantly between the NP and other reproductive stages. The NP group showed a significant increase in the levels of lipids and lipid-like molecules, including 11-Dehydro-thromboxane B2, 20-Carboxy-leukotriene B4, 20-hydroxy LTB4, 9S-hydroxy-11,15-dioxo-5Z,13E-prostadienoic acid, PGA2, (5Z)–(15S)-11alpha-Hydroxy-9,15-dioxoprostanoate, 5(S)-HETE, and 15-Hydroxy-5,8,11,13-eicosatetraenoic acid. These findings align with previous research by Burdge et al. [81] and Min et al. [82], which reported lower levels of AA in pregnant mice and humans. In the NP stage, compared to the other reproductive stages, there was a significant upregulation of lipids and lipid-like molecules, including traumatin, trauma acid, 9(S)-HpOTrE, 9-Oxo-nonanoic acid, (9Z,11E,13S,15Z)-13-Hydroxyoctadeca-9,11,15-trienoic acid, and stearidonic acid, which play an important role in the alpha-linolenic acid metabolism exhibits a significant change in alpha-linolenic acid metabolism, aligning with the study by Jungheim et al. [83] which revealed that women with higher serum alpha-linolenic acid levels had a lower pregnancy chance. The high abundance of these metabolites during NP reveals that there is less chance of pregnancy in the captive YFP. As compared with the NP, the EP and MP had much higher concentrations of lipids and lipids-like molecule such as 2-isopropylmalic acid. These lipids also significantly altered the production of valine, leucine, and isoleucine, which is also altered in humans during pregnancy [84]. Lipids and lipid-like molecules (LysoPC(18:3(6Z,9Z,12Z)/0:0), LysoPC(14:1(9Z)/0:0), PA(20:5(5Z,8Z,11Z,14Z,17Z)/20:5(5Z,8Z,11Z,14Z,17Z)), PS(18:4(6Z,9Z,12Z,15Z)/22:4(7Z,10Z,13Z,16Z)), CDP-DG(18:2(9Z,11Z)/i-12:0) and CDP DG(22:6(4Z,7Z,10Z,13Z,16Z,19Z)/18:2(9Z,12Z))) significantly higher in the LL group compared to NP, and they significantly altered the glycerophospholipid metabolism, which is known to decrease before calving and increase after [85]. These metabolites may play an important role in maternal health and fetal growth during the lactation stage of the captive YFP.

The current study found a strong correlation between fecal metabolites and fecal microbiota, using Spearman correlation analysis to evaluate the metabolite-microbial connections. Positive correlations were found between the cholic acid and 12-hydroxy-3-oxo-5beta-cholan-24-oic acid with the *Terrisporobacter*, *unclassified Clostridiaceae*, *Clostridium*, *Clostridium_sensu_stricto_13*, and *unclassified Clostridia*, which may have a significant effect on the captive YFP’s pregnancy. Bile acids are the main chemical constituent of bile and are a class of cholesterol metabolites. They proceed via enterohepatic circulation, transmembrane transport, and synthesis, which allow dietary lipids and fat-soluble vitamins to be micellized and absorbed. In addition, they function as signaling molecules that regulate inflammation and metabolism [86, 87]. As compared to the non-pregnancy stages, the current study showed significant increases in the levels of bile acid derivatives, including cholic acids, taurochenodeoxycholic acid, lithocholic acid glycine conjugate, and sulfolithocholic acid, during pregnancy. These findings were consistent with the increase in bile acids during human pregnancies [87, 88]. This increase may be due to the suppression of the Farnesoid X receptor (FXR), which has been linked to an upregulation of the expression of the human sterol 12α-hydroxylase gene (Cyp8b1), a gene that FXR regulates and is implicated in the manufacture of cholic acid [89]. Unconjugated bile acids assist in the digestion and absorption of fat and alter throughout pregnancy [87].

In addition, the limitations of this study include the use of two animals and the small sample size. Further research is needed to monitor microbe shifts in many animals while increasing sample sizes and understanding the mechanisms of cross-talk between the fecal microbiota, metabolites, host metabolism, and its function and significance in host health.

## Conclusion

The study examined the association of fecal metabolites and gut microbiota in captive YFP during reproductive stages. Firmicutes, which play an important role in energy metabolism, remained stable and uniform throughout the reproductive period. The genus *Clostridium*, essential for the breakdown of cellulose, was found to be considerably more prevalent in the EP than in the other groups and is also believed to be more abundant in pregnant pandas [61, 90]. *Clostridium sensu stricto 1* was abundant in the LL as compared to the other stages due to their high energy requirements and high food diet. In addition, there were significant variations in the host fecal metabolome. Lipids and lipid-like molecules, such as galactosylglycerol, which are crucial for the metabolism of carbohydrates and galactose, as well as fetal development, were significantly higher in the pregnant stage as compared to the other stages and reported to be higher in pregnant humans [71]. Significant variations were observed in the KEGG pathways, including vitamin absorption and digestion, nucleotide metabolism, and the metabolism of valine, leucine, and isoleucine, phospholipid metabolism, linoleic acid, arachidonic acid, alpha-linolenic acid, purine metabolism, and glucose metabolism in different reproductive stages. Additionally, the current study revealed a significant correlation between changes in the YFP fecal microbiome and changes in host metabolite. This study provides a reference and important information for improving and managing captive YFP during d/f reproductive stages. Further validation is needed to confirm the microbiota-metabolite network’s role in fetal growth and maternal adaptation, potentially by transplanting fecal microbiota into germ-free mice.

## Supplementary Material

Supplementary_file_Table_ioae123

Table_S2_ioae123

Table_S3_ioae123

Table_S4_ioae123

Supplementary_file_Figures_ioae123

## Data Availability

The data underlying this article is available in the article and its supplementary material. The raw sequencing data has been deposited in the NCBI SRA repository under the accession number PRJNA1129335.
