# Supplementary material for: Physiological function of gut microbiota and metabolome on successful pregnancy and lactation in the captive Yangtze finless porpoise (Neophocaena asiaeorientalis asiaeorientalis)
Source: Biol Reprod. 2024 Aug 13;111(6):1249–61. doi: 10.1093/biolre/ioae123 (PMC11647103; doi:10.1093/biolre/ioae123)
Supplement: Supplementary_file_Table_ioae123 [file supplementary_file_table_ioae123.docx]

**Table S1:** Alpha-diversity of the gut microbiomes in different reproductive stages of the captive YFP in this study.

|  | **NP** | **EP** | **MP** | **LL** | **P-value** |
| --- | --- | --- | --- | --- | --- |
| **Ace** | 95.79 | 76.88 | 78.58 | 62.74 | 0.1574 |
| **Chao** | 92.44 | 73.72 | 70.56 | 60.17 | 0.1609 |
| **Sobs** | 0.9997 | 0.9998 | 0.9998 | 0.9999 | 0.2593 |
| **Shannon** | 1.328 | 1.905 | 1.561 | 1.925 | 0.08426 |
| **Coverage** | 0.3811 | 0.234 | 0.3215 | 0.2149 | 0.2276 |
| **Simpson** | 86.33 | 69.33 | 62.67 | 57 | 0.1609 |
