# Supplementary material for: Physiological function of gut microbiota and metabolome on successful pregnancy and lactation in the captive Yangtze finless porpoise (Neophocaena asiaeorientalis asiaeorientalis)
Source: Biol Reprod. 2024 Aug 13;111(6):1249–61. doi: 10.1093/biolre/ioae123 (PMC11647103; doi:10.1093/biolre/ioae123)
Supplement: Supplementary_file_Figures_ioae123 [file supplementary_file_figures_ioae123.docx]

**Figure S1: (a)** Microbiota rarefaction curve generated using the Shannon index with samples of NP, EP, MP, and LL. **(b)** Venn diagram showing the overlapping numbers of OTUs between the d/f reproductive stages in the captive YFP.

**Figure S2:** Alpha diversity of gut microbiota from captive Yangtze finless porpoise. (a) ACE index at OTU level. (b) Chao index at OTU level. (c) Shannon index at OTU level. (d) Simpson index at OTU level. Significant differences between different reproductive groups were tested by the Wilcoxon rank-sum test.

**Figure S3:** The predicted KEGG pathways within different reproductive stages in the captive YFP. NP, non-pregnancy; EP, early pregnancy; MP, mid-pregnancy; LL, lactating stage. The X-axis represents the sample name; the Y-axis shows the function name of the KEGG pathway; the changes of different functional abundances in samples are displayed by color block; the red represents a higher abundance; the blue represents a lower abundance.

**Figure S4:** Screening of potential differential metabolites in the captive YFP. (a-d) The differential metabolites for NP-EP, NP-MP, NP-LL, and EP-LL, respectively, are represented by a volcano. The metabolite expression difference between the two groups is shown by the abscissa, or log2FC, which is the multiple change value. The statistical P-value of metabolite differential expression, or -log10, is represented by the ordinate. The significance of the expression difference increases with increasing value.

**Figure S5:** KEGG pathway classification: metabolites detected and annotated in the captive YFP. The x-axis represents the number of metabolites identified and the y-axis represents level-2 terms of the KEGG pathway.
